# Association of healthy eating index and self-rated health in adults living in Tehran: a cross-sectional study

**DOI:** 10.1186/s12889-024-18568-w

**Published:** 2024-04-22

**Authors:** Bahareh Jabbarzadeh-Ganjeh, Kurosh Djafarian, Sakineh Shab-Bidar

**Affiliations:** 1 Department of Community Nutrition, School of Nutritional Sciences and Dietetics, Tehran University of Medical Sciences (TUMS), No 44, Hojjat-dost Alley, Naderi St., Keshavarz Blvd, 14167-53955 Tehran, 14155/6117, Iran; 2 Department of Clinical Nutrition, School of Nutritional Sciences and Dietetics, Tehran University of Medical Sciences (TUMS), 14167-53955 Tehran, Iran; 3 Sports Medicine Research Center, Neuroscience Institute, Tehran University of Medical Sciences (TUMS), Tehran, Iran

**Keywords:** Diet, healthy, Eating, Self-assessment, Self-rated health

## Abstract

**Background:**

Self-rated health (SRH) has been identified in many studies as a valid predictor of mortality and healthcare utilization. There is limited research on SRH and dietary intake. This study aimed to investigate the association between healthy eating index (HEI) and SRH in adults living in Tehran.

**Methods:**

This cross-sectional study was carried out among 850 adult men and women aged 20–59 years who visited health centers in Tehran from 2021 to 2022. Dietary intake was assessed using a validated and reliable semiquantitative food frequency questionnaire with 168 food items, and SRH was assessed with one question: “In general, how do you rate your health?“. We categorized SRH into excellent/very good, good, and fair/poor. In the descriptive statistics part, we used mean ± standard deviation or number (ratio) for quantitative and qualitative variables, respectively. The chi-squared test and one-way analysis of variance were used to calculate the percentage and mean for demographic characteristics across tertiles of SRH. An analysis of covariance was used to compare the means of energy, macronutrients, the HEI, and its component variables across the tertiles of SRH.

**Results:**

The final sample included 795 participants (68.2% female; mean ± standard deviation age: 44.81 ± 10.62 years) whose 40% reported excellent/very good SRH, and 30% reported good and fair/poor SRH separately. There was no association between body mass index, physical activity, education, health status, smoking, and sleep duration with SRH. After adjustment, the total HEI score and its component scores did not differ across the tertiles of SRH status. However, participants with good SRH had a higher intake of total energy (mean difference (MD): 180.33 Kcal, P value < 0.001), total fat (MD: 8.15 gr, P value = 0.002), and total carbohydrates (MD: 20.18 gr, P value = 0.004) than those with fair/poor SRH.

**Conclusion:**

According to our findings, fair/poor SRH was associated with a lower consumption of total energy, total fat, and total carbohydrates in Iranian adults. Additional observational studies would be necessary to clarify these findings.

## Introduction

Self-rated health (SRH) is one of the most commonly evaluated health conceptions in population-based and clinical studies [1, 2]. SRH asks people to rate their general health qualitatively via a short question [3]. It has been identified in many studies as a strong predictor of mortality and might predict the incidence of diseases [4]. Some studies have shown that poor SRH status could predict greater healthcare utilization and diminish physical performance in the adult population [3, 5]. The total cost of physical or psychological disorders and illnesses, including healthcare expenses and lost economic productivity, amounts to trillions [6]. Therefore, SRH as a screening tool [2] might be helpful to lower the healthcare budget. SRH affects the healthy behaviors of people. For older adults, SRH is a retrospective health history assessment and is, therefore, more indicative of their health status than many blood markers [7].

In the past, nutritional epidemiology mostly focused on the relationship between diseases and specific nutrients, such as vitamins, or specific food groups, such as vegetables and fruits. However, currently, more studies are focused on dietary patterns and evaluating the quality and variety of the whole diet [5]. Based on dietary guidelines, the diet plan should be low-fat, rich in fruits and vegetables, and generally have a high nutrient density [5]. There are different ways to assess diet quality, such as the food frequency questionnaire (FFQ), healthy eating index (HEI), alternative healthy eating index (AHEI), etc [5, 8, 9]... The HEI was created on dietary guidelines for Americans, and the scoring was based on adequacy components (foods to eat more of for good health) and moderation components (foods to limit for good health) [10]. Both HEI and AHEI scores ranged from 0 to 100, where a higher score presents a healthier diet [11]. A cross-sectional study in Tehran recently estimated the mean HEI score was 52.5, while it was 62.3 in 2001 [12, 13]. A systematic review assessed the diet quality using AHEI, and the worldwide mean score was 40.3 among 185 countries from 1990 to 2018. Another systematic review of cohort studies found an association between higher scores of HEI and AHEI with lower risk of all-cause mortality (20%), cardiovascular disease (20%), cancer (14%), type 2 diabetes (19%), and neurodegenerative disease (18%) [14]. One study reported that those who consumed equal to or less than two servings of fruits and vegetables per day or consumed high-fat foods mostly had poor/fair SRH scores [15]. Another cross-sectional study revealed a significant and negative association between overweight and low physical activity with SRH [16].

To date, the status of SRH has not been investigated in Iran. Furthermore, studies exploring the relationship between SRH and the quality of diet, particularly HEI, are scarce. We wanted to determine whether having a better SRH can be associated with a better healthy diet. Conducting this study will help us to have a better understanding of SRH and self-assessment among Iranians. Therefore, this study aimed to determine the association between healthy eating index and self-rated health in adults living in Tehran.

## Methods

### Study design

The study was a cross-sectional population-based survey of those aged 20–59 years from 2021 to 2022. The data were collected from 850 healthy adult men and women through two-stage cluster sampling. In the first stage, a simple random sample was selected within 25 healthcare centers across five different geographic areas of Tehran, and a convenient sampling method was used for the second stage. People who visited health centers in Tehran and met the inclusion criteria were informed about the implementation and objectives of the study via informed consent forms. The research was approved by the Tehran University of Medical Science Human Research Ethics Committee (IR.TUMS.MEDICINE.REC.1401.604).

### Data collection

A demographic questionnaire was used for general information through face-to-face interviews. It included age (year), sex (male/female), education level (illiterate/under diploma/diploma/university), occupation (employed/unemployed), marital status (single/married), smoking (yes/quit smoking/no), health status (healthy/one disease/comorbidity), and sleep duration.

### Dietary intake assessment

We used a validated and reliable semiquantitative FFQ with 168 food items for each participant to assess their dietary intake. The nutritionist asked about FFQs from the participants through face-to-face interviews. The macro- and micronutrient intake were analyzed using Iranian-designed Nutrition IV Software (First Database, San Bruno, CA).

HEI was calculated based on predetermined criteria by the United States Department of Agriculture [17]. The 2015 version of this index has nine components related to adequacy and four related to moderation. The total score is the sum of the score of adequacy components (i.e. foods to eat more of for good health) and moderation components (i.e. foods to limit for good health). The HEI scores ranged from 0 to 100, where a higher score presents a healthier die [18]. The adequacy part includes the following: (1) Total fruit (includes fruit juice), (2) Whole fruits (all forms except fruit juice), (3) Total vegetables (includes any beans and peas), (4) Greens and beans (includes any beans and peas), (5) Whole grains, (6) Dairy (includes all milk products, such as fluid milk, yogurt, and cheese, and fortified soy beverages), (7) Total protein foods (beans and peas are included here (and not with vegetables) when the Total Protein Foods standard is otherwise not met), (8) Seafood and plant proteins (includes seafood, nuts, seeds, soy products (other than beverages) as well as beans and peas if they counted as Total Protein Foods), and (9) Fatty acids (ratio of poly- and monounsaturated fatty acids to saturated fatty acids). The moderation components consist of (1) refined grains, (2) sodium, (3) added sugars, and (4) saturated fats [18].

### Self-rated health assessment

SRH was assessed by asking one question, “In general, how do you rate your health?“. The answers include excellent, very good, good, fair, and poor [19]. For this study, we combined the “excellent, very good” responses as one subgroup and “fair, poor” responses as another. Therefore, SRH responses were categorized into excellent/very good, good, and fair/poor. This method aligns with other studies that have used the SRH status question [2, 20] and makes a better differentiation between positive and negative responses [21].

### Physical activity

We used the short form of the international physical activity questionnaire, validated for the Iranian population [22]. Participants were questioned about the time spent on vigorous, moderate, and walking activities within the last seven days. The physical activity score was calculated based on the metabolic equivalent minutes per week (MET-minutes/week). At last, the physical activity level is categorized into low (< 600 MET-min/week), moderate (600–3000 MET-min/week), and high levels (> 3000 MET-min/week) [23].

### Assessment of blood pressure

Blood pressure was measured twice by a digital sphygmomanometer (Beurer, BC 08, Germany) after at least 10–15 min of rest. An average of two blood pressures was reported for each person.

### Anthropometric measurements

The participant’s height without shoes was measured using a wall stadiometer with a sensitivity of 0.1 cm (Seca, Germany). Weight was evaluated by a digital scale (808 Seca, Germany) with an accuracy of 0.1 kg with minimum clothes on. Body mass index (BMI) was calculated by dividing the weight (kg) by the square of the height (m) [24]. Based on the WHO, the BMI cut-off points for determining underweight, normal weight, overweight, and obesity are < 18.5, 18.5–24.9, 25-29.9, and ≥ 30, respectively [25]. Waist (WC) and hip (HC) circumferences were measured with a flexible nonelastic metric tape. WC was measured between the lowest rib and the Iliac crest during exhalation, while HC was at the point that yielded the maximum diameter over the buttocks [24]. The waist-to-hip ratio (WHR) was calculated by dividing the WC (cm) by HC (cm) [26]. The waist-to-height ratio (WHtR) was computed by dividing the WC (cm) by height (cm) [27]. We applied a single nutritionist performing all the measurements to reduce the odds of subjective errors.

### Statistical analysis

The general characteristics of the participants are displayed as the mean and standard deviation or number and percent. We categorized SRH into excellent/very good, good, and fair/poor. The normality test of the data was through the Kolmogorov‒Smirnov test and the Q‒Q plot to determine the normal distribution of the data. The chi-squared test and one-way analysis of variance (ANOVA) were calculated as the percentage and mean for demographic characteristics across tertiles of SRH. To compare the means of energy, macronutrients, the HEI, and its component variables across the tertiles of SRH, we applied an analysis of covariance (ANCOVA), adjusting for age, sex, education, occupation, marital status, smoking status, health status, physical activity, and BMI. All analyses were performed with SPSS (SPSS Inc., version 26) software. A p-value less than 0.05 accounted for a significant difference.

## Results

Based on Table 1, the mean ± standard deviation of the participant’s age was 44.81 ± 10.62 years old. Of 850 participants, 17 were excluded due to underreporting, extreme values for protein and total fat intake, and 38 due to lack of information. The final sample included 795 participants, and 542 were female. In total, 40% of the population reported excellent/very good SRH, and 30% reported good and fair/poor SRH separately.

**Table 1:** General characteristics of study participants

Characteristics	All (n = 795)	Men (n = 253)	Women (n = 542)	P value
**Age (year)**	44.81 ± 10.62	45.43 ± 9.68	44.51 ± 11.02	0.237
**Height (cm)**	162.89 ± 8.88	170.71 ± 6.97	159.23 ± 7.14	**< 0.001**
**Weight (Kg)**	73.70 ± 13.48	80.95 ± 13.88	70.32 ± 11.87	**< 0.001**
**BMI (kg/m** ^**2**^ **)**	27.79 ± 4.67	27.73 ± 4.04	27.83 ± 4.94	0.761
**WC (cm)**	92.14 ± 12.22	95.57 ± 11.44	90.55 ± 12.25	**< 0.001**
**WHR**	0.88 ± 0.08	0.93 ± 0.07	0.86 ± 0.08	**< 0.001**
**WHtR**	0.57 ± 0.08	0.56 ± 0.07	0.57 ± 0.09	0.087
**SBP (mmHg)**	119.72 ± 22.66	121.27 ± 23.04	119 ± 22.47	0.188
**DBP (mmHg)**	78.30 ± 14.07	78.90 ± 14.43	78.02 ± 13.91	0.417
**Self-rated health**				**0.052**
Excellent/very good	318 (40)	96 (37.95)	222 (40.96)	
Good	239 (30.06)	67 (26.48)	172 (31.73)	
Fair/poor	238 (29.94)	90 (35.57)	148 (27.31)	
**Education**				**0.044**
Illiterate	67 (8.43)	15 (5.93)	52 (9.60)	
Under diploma	211 (26.54)	77 (30.43)	134 (24.72)	
Diploma	242 (30.44)	66 (26.09)	176 (32.47)	
University	275 (34.59)	95 (37.55)	180 (33.21)	
**Occupation**				**< 0.001**
Employed	206 (25.91)	102 (40.32)	104 (19.19)	
Unemployed	589 (74.09)	151 (59.68)	438 (80.81)	
**Marriage**				**< 0.001**
Single	147 (18.49)	24 (9.49)	123 (22.69)	
Married	648 (81.51)	229 (90.51)	419 (77.31)	
**Smoking**				**< 0.001**
Not smoking	720 (90.57)	204 (80.63)	516 (95.20)	
Quit smoking	32 (4.02)	16 (6.33)	16 (2.95)	
Smoking	43 (5.41)	33 (13.04)	10 (1.85)	
**Physical activity (MET/min/wk)**				0.394
Low	504 (63.40)	155 (61.26)	349 (64.39)	
Moderate	291 (36.60)	98 (38.74)	193 (35.61)	
High	0	0	0	
**Diabetes**	68 (8.56)	26 (10.32)	42 (7.75)	0.229
**CVD**	46 (5.79)	7 (2.77)	39 (7.20)	**0.013**
**Hypertension**	127 (15.97)	39 (15.42)	88 (16.24)	0.768
**Dyslipidemia**	111 (13.96)	36 (14.23)	75 (13.84)	0.882
**Cancer**	3 (0.38)	1 (0.40)	2 (0.37)	0.342

Data are shown as mean ± standard deviation for quantitative data, and number (percent) for qualitative data

Results are from independent t-test for quantitative data, and chi-square test of association for qualitative data

BMI, body mass index; WC, waist circumference; WHR, waist to hip ratio; WHtR, waist to height ratio; SBP, systolic blood pressure; DBP, diastolic blood pressure; MET/min/wk, metabolic equivalent-minutes per week; CVD, cardiovascular disease

Table 2 shows the frequency and the mean of some demographic characteristics across tertiles of SRH. There was no association between BMI, physical activity, education, health status, smoking, or sleep duration, and SRH.

**Table 2:** The percentage for demographic characteristics across tertiles of self-rated health

	Total (n = 795)	Excellent and Very Good (n = 318)	Good (n = 239)	Fair and Poor (n = 238)	P value
**BMI**					0.876
BMI < 18.5	12 (1.51)	4 (1.26)	4 (1.67)	4 (1.68)	
18.5 ≤ BMI < 25	208 (26.16)	80 (2516)	68 (28.45)	60 (25.21)	
25 ≤ BMI < 30	349 (43.90)	137 (43.08)	107 (44.77)	105 (44.11)	
BMI ≥ 30	226 (28.43)	97 (30.50)	60 (25.11)	69 (29)	
**Physical activity (MET/min/wk)**					0.338
Low	504 (63.40)	211 (66.35)	149 (62.34)	144 (60.50)	
Moderate	291 (36.60)	107 (33.65)	90 (37.66)	94 (39.50)	
**Education**					0.906
Illiterate	67 (8.43)	26 (8.18)	20 (8.37)	21 (8.82)	
Under diploma	211 (26.54)	86 (27.04)	59 (24.69)	66 (27.73)	
Diploma	242 (30.44)	93 (29.25)	81 (33.89)	68 (28.57)	
University	275 (34.59)	113 (35.53)	79 (33.05)	83 (34.88)	
**Health status**					0.350
Healthy	520 (65.41)	212 (66.67)	161 (67.36)	147 (61.76)	
One disease	145 (18.24)	52 (16.35)	47 (19.67)	46 (19.33)	
Comorbidity	130 (16.35)	54 (16.98)	31 (12.97)	45 (18.91)	
**Smoking**					0.548
Not smoking	720 (90.57)	285 (89.62)	223 (93.31)	212 (89.08)	
Quit smoking	32 (4.03)	14 (4.40)	7 (2.93)	11 (4.62)	
Smoking	43 (5.41)	19 (5.97)	9 (3.77)	15 (6.30)	
**Sleep duration**	6:47 ± 1:15	6:45 ± 1:18	6:49 ± 1:09	6:48 ± 1:16	0.772

Data are shown as number (percent) for qualitative data, and mean ± standard deviation for quantitative data

Results are from chi-square test of association for qualitative data, and one-way analysis of variance (ANOVA) for quantitative data

BMI, body mass index; MET/min/wk, metabolic equivalent-minutes per week

Table 3 indicates the multivariate-adjusted means of the HEI and its component scores across tertiles of SRH status. The results from the Tukey post hoc test showed that participants with good SRH compared with fair/poor SRH had significant differences in total energy consumption (mean difference (MD): 180.33 Kcal, P value < 0.001), total carbohydrate (MD: 20.18 gr, P value = 0.004), and total fat intake (MD: 8.15 gr, P value = 0.002). Additionally, those with good SRH had lower scores for Total Vegetable (P value = 0.058), Greens and Beans (P value = 0.059), and Dairy (P value = 0.042) compared with participants with fair/poor SRH. However, after adjusting for confounders, the marginal and significant differences were all gone.

**Table 3:** Multivariate adjusted means for healthy eating index and component scores across tertiles of self-rated health status among Iranian adults

	Total (n = 795)	Fair and Poor (n = 238)	Good (n = 239)	Excellent and Very Good (n = 318)	P ANOVA	P ANCOVA
**Total Energy (kcal)**	2152.05 ± 519.67	2065.53 ± 533.30	2245.86 ± 502.47	2146.30 ± 511.79	**0.001**	**< 0.001**
**Total Carbohydrate (gr)**	328.17 ± 90.12	322.35 ± 98.19	342.53 ± 81.89	321.73 ± 88.76	**0.013**	**0.004**
**Total Protein (gr)**	82.50 ± 31.81	81.95 ± 33.36	85.69 ± 31.82	80.52 ± 30.50	0.157	0.091
**Total Fat (gr)**	77.03 ± 35.13	74.75 ± 33.40	82.90 ± 37.66	74.33 ± 33.98	**0.008**	**0.002**
**Total HEI score**	52.31 ± 11.48	52.47 ± 11.1	51.50 ± 11.79	52.79 ± 11.53	0.408	0.246
**Adequacy**						
Total Fruits score	3.12 ± 1.54	3.23 ± 1.57	2.95 ± 1.55	3.15 ± 1.50	0.115	0.261
Whole Fruit score	3.02 ± 1.27	3.05 ± 1.24	2.89 ± 1.32	3.08 ± 1.25	0.171	0.224
Total Vegetable score	3.10 ± 1.49	3.29 ± 1.49	2.99 ± 1.48	3.03 ± 1.48	**0.058**	0.206
Greens and Beans score	2.87 ± 1.38	3.04 ± 1.33	2.74 ± 1.40	2.84 ± 1.40	**0.059**	0.197
Whole Grain score	6.88 ± 3.19	7.25 ± 2.97	6.63 ± 3.35	6.78 ± 3.20	0.079	0.193
Dairy score	5.78 ± 3.05	6.15 ± 3.06	5.45 ± 2.99	5.75 ± 3.06	**0.042**	0.116
Total Protein score	3.17 ± 1.59	3.30 ± 1.58	3.05 ± 1.52	3.16 ± 1.63	0.230	0.497
Seafood and Plant Proteins score	2.50 ± 1.14	2.62 ± 1.12	2.38 ± 1.11	2.49 ± 1.18	0.084	0.176
Fatty Acids score	4.95 ± 3.29	4.62 ± 3.16	5.00 ± 3.33	5.15 ± 3.35	0.168	0.107
**Moderation**						
Refined Grains score	2.44 ± 3.48	2.21 ± 3.34	2.88 ± 3.67	2.29 ± 3.41	0.069	0.072
Sodium score	3.58 ± 3.89	3.52 ± 3.84	3.46 ± 3.79	3.72 ± 4.01	0.699	0.442
Added Sugars score	4.57 ± 3.13	4.18 ± 3.19	4.77 ± 30.6	4.72 ± 3.12	0.070	0.413
Saturated Fats score	6.32 ± 3.93	6.00 ± 4.02	6.23 ± 4.02	6.63 ± 3.80	0.168	0.133

Data are shown as mean ± standard deviation

Adjusting for age, sex, education, occupation, marital status, smoking status, physical activity, health status, and body mass index

ANOVA, one-way analysis of variance; ANCOVA, analysis of covariance; HEI, healthy eating index

## Discussion

In this cross-sectional study, we aimed to investigate the association between a healthy eating index (HHEI) and self-rated health (SRH) in adults living in Tehran. Our study found that 40% (318 participants) reported excellent/very good SRH, 30.1% (239 participants) reported good SRH, and 29.9% (238 participants) reported fair/poor SRH. There were no statistically significant associations between BMI, physical activity, education, health status, smoking, or sleep duration, and SRH. After adjustment, the total HEI score and its component scores did not differ across the tertiles of SRH status. However, participants with good SRH had a higher intake of total energy, total fat, and total carbohydrates than those with fair/poor SRH.

Two studies involving younger populations reported similar findings. A cross-sectional study among 1504 US adolescents found no significant association between HEI score and SRH. However, further analysis revealed specific dietary differences: those with excellent-good SRH had a higher vegetable score, while those with fair/poor SRH had a higher total fat intake score [3]. A cohort study conducted from 2003 to 2012 on 953 German participants also found no significant association between SRH and those with high healthy nutrition scores and below-average scores in the physical activity and media use index [28]. The studies suggest this might be due to developmental differences and potentially limited awareness of healthy eating habits in younger individuals [3, 28].

Several studies support the association between unhealthy lifestyle factors and poorer SRH. The Spanish DiSA-UMH study found that poorer SRH was linked to lower adherence to the Mediterranean diet, lower physical activity levels, excess weight, and smoking among university students [29]. Similarly, studies by Zarini et al. and Collins et al. linked fair/poor SRH to higher fat intake [5, 15], lower fruit and vegetable intake, and lower physical activity, particularly among females [15]. These findings align with our null findings for BMI and smoking, as reported in another study conducted in a rural Greek population [2].

Some studies reported positive associations between HEI and SRH, contrasting with our findings. Vaudin et al. observed a link between better SRH and a more favorable HEI score in older adults [20]. Additionally, studies in rural populations found associations between healthier diets and better SRH [1, 2], while lower education and chronic diseases were linked to poorer SRH [2]. A large survey [30] also reported associations between poor sleep, physical inactivity, and poor diet quality with higher odds of poor SRH. However, it’s important to note that the participants in this survey had reported “good” SRH earlier.

Possible explanations for these contrasting findings include:

Population differences: The studies with contrasting findings involved different age groups, health statuses, and potentially socioeconomic backgrounds compared to our study population.

Health awareness: Individuals with chronic diseases might have higher health awareness due to more frequent medical consultations, potentially leading to healthier dietary choices [15].

Confounding factors: Many variables beyond those we adjusted for in our study, such as socioeconomic status [31, 32] and mental health [32], can influence SRH.

This study has some limitations. First, some confounders, such as social well-being, were not adjusted. Second, the cross-sectional design and the lack of significant associations between HEI and SRH might mirror low power due to the small sample size in this analysis. Our study also has some strengths. The strengths of the current observational study include a sample representative of Tehran’s general population, the first study in Iran around this field, using the latest version of the HEI, a gold standard tool for assessing usual food intake (FFQ), and the inclusion of a large number of covariates.

## Conclusion

This is the first attempt to relate SRH status to HEI in healthy Iranian adults. The total HEI score did not vary by SRH status. In detail, those with good SRH had a higher intake of total energy, total fat, and total carbohydrates than those with fair/poor SRH. Additional observational studies are needed to clarify these findings.

## Data Availability

The datasets analyzed during the current study are available from the corresponding author on reasonable request.

## Abbreviations

SRH: self-rated health
FFQ: food frequency questionnaire
HEI: healthy eating index
BMI: Body mass index
WC: Waist circumference
HC: hip circumferences
WHR: waist-to-hip ratio
WHtR: waist-to-height ratio
SD: standard deviation
ANOVA: one-way analysis of variance
ANCOVA: analysis of covariance
MD: mean difference
